# Chitinophaga pollutisoli sp. nov., isolated from contaminated sediment

**DOI:** 10.1099/ijsem.0.006447

**Published:** 2024-07-04

**Authors:** Dae Seung Lee, Jae Kyeong Lee, Dong Min Han, Ju Hye Baek, Che Ok Jeon

**Affiliations:** 1 Department of Life Science, Chung-Ang University, Seoul 06974, Republic of Korea

**Keywords:** *Bacteroidota*, *Chitinophaga pollutisoli*, new taxa, waste landfill soil

## Abstract

A Gram-stain-negative, yellow-pigmented, and facultatively aerobic bacterium, designated strain GPA1^T^, was isolated from plastic waste landfill soil in the Republic of Korea. The cells were non-motile short rods exhibiting oxidase-negative and catalase-positive activities. Growth was observed at 15–40 °C (optimum, 30 °C), at pH 6.0–9.0 (optimum, pH 7.0–8.0) and in the presence of 0–2.5 % (w/v) NaCl (optimum, 0 %). Menaquinone-7 was the sole respiratory quinone, and iso-C_15 : 0_, C_16 : 1_* ω*5*c*, and iso-C_17 : 0_ 3-OH were the major cellular fatty acids (>10 % of the total fatty acids). Phosphatidylethanolamine was identified as a major polar lipid. Phylogenetic analyses based on 16S rRNA gene sequences and 120 concatenated marker protein sequences revealed that strain GPA1^T^ formed a distinct lineage within the genus *Chitinophaga*. The genome of strain GPA1^T^ was 6078 kb in size with 53.8 mol% G+C content. Strain GPA1^T^ exhibited the highest similarity to *Chitinophaga rhizosphaerae* T16R-86^T^, with a 98.6 % 16S rRNA gene sequence similarity, but their average nucleotide identity and digital DNA–DNA hybridization values were 82.5 and 25.9 %, respectively. Based on its phenotypic, chemotaxonomic, and phylogenetic characteristics, strain GPA1^T^ represents a novel species of the genus *Chitinophaga*, for which the name *Chitinophaga pollutisoli* sp. nov. is proposed. The type strain is GPA1^T^ (=KACC 23415^T^=JCM 36644^T^).

## Introduction

The genus *Chitinophaga* was first proposed by Sangkhobol and Skerman, with *Chitinophaga pinensis*, capable of chitin degradation, as the type species [1]. Subsequently, the genus underwent emendation and was established as the type genus of the family *Chitinophagaceae* within the phylum *Bacteroidota* [2]. As of April 2024, this genus comprises 47 species with validly published names and eight species with invalidly published names (https://lpsn.dsmz.de/genus/chitinophaga), isolated from diverse environmental habitats including soil [3,5], an air conditioner condensate drain line [6], activated sludge [7], plant root [8], arsenic-contaminated soil [9], and vermicompost [10]. Members of the genus *Chitinophaga* are Gram-stain-negative, non-spore-forming, aerobic rod-shaped bacteria containing menaquinone-7 (MK-7) as the major respiratory quinone, iso-C_15 : 0_, C_16 : 1_* ω*5*c*, and iso-C_17 : 0_ 3-OH as the major cellular fatty acids, and phosphatidylethanolamine (PE) as a major polar lipid [3,10]. The genus *Chitinophaga* was initially coined to denote bacteria capable of chitin degradation, but interestingly, it has been observed that most *Chitinophaga* species, except *C. pinensis* and *Chitinophaga filiformis*, lack this ability [1,3,11]. In this study, we isolated a putative novel strain affiliated with the genus *Chitinophaga* from an enriched culture using phthalic acid (PA) and soil sediment, and its taxonomic characteristics were assessed using a polyphasic approach.

## Strain isolation

Strain GPA1^T^ was isolated from an enriched culture using PA and contaminated soil sediment in minimal salts basal (MSB) medium [12], following previously described methods [13,14]. To enrich PA-degrading bacteria, approximately 10 g sediment collected from a stream within an industrial complex in Incheon, Republic of Korea (37° 36′ 10.4″ N 126° 36′ 50.9″ E), was added to a cotton-plugged 500 ml Erlenmeyer flask containing 0.3 g PA as a sole carbon and energy source in 100 ml MSB medium. The flask was shaken at 180 r.p.m. on a rotary shaker at 25 °C, and the culture was transferred (1 : 10) to fresh MSB medium containing 0.3 g PA every 2 weeks for three successive transfers. To isolate bacterial strains from the enriched culture, the final culture was serially diluted in PBS (137 mM NaCl, 2.7 mM KCl, 10 mM Na_2_HPO_4_, 2 mM KH_2_PO_4_, pH 7.2) and aliquots of each dilution were spread onto Reasoner's 2A (R2A) agar (MBcell). The agar plates were then aerobically incubated at 25 °C until colonies were visible.

Colonies grown on R2A agar were subjected to PCR amplification of the 16S rRNA genes using universal primers 27F (5′-AGAGTTTGATCMTGGCTCAG-3′) and 1492R (5′-TACGGYTACCTTGTTACGACTT-3′) [14]. Subsequently, the PCR products were double-digested with *HaeIII* and *HhaI*, followed by analysis using 2 % agarose gel electrophoresis. PCR products exhibiting distinct fragment patterns were partially sequenced with the universal primer 340F (5′-CCTACGGGAGGCAGCAG-3′) [14]. The obtained sequences were compared with those of all type strains of validly published species on the EzBioCloud server (http://www.ezbiocloud.net/) [15]. Based on this analysis, a potential novel strain, GPA1^T^, belonging to the genus *Chitinophaga*, was selected for further taxonomic characterization. Strain GPA1^T^ was routinely cultured aerobically on R2A agar at 30 °C for 2 days and preserved at −80 °C in R2A broth (MBcell) supplemented with 15 % (v/v) glycerol. Reference strains *Chitinophaga rhizosphaerae* KACC 18790^T^, *Chitinophaga caseinilytica* KACC 19118^T^, and *Chitinophaga deserti* KCTC 62443^T^ were acquired from their respective collection centres and utilized for comparison of genomic characteristics, phenotypic properties, and fatty acid compositions.

## Phylogeny based on 16S rRNA gene sequences

The 16S rRNA amplicon of strain GPA1^T^, amplified by the 27F and 1492R primers, was further sequenced using the universal primers 518R (5′-ATTACCGCGGCTGCTGG-3′) and 805F (5′-GATTAGATACCCTGGTAGTC-3′) [14]. The sequences obtained from the 340F, 518R, and 805F primers were assembled to generate a nearly complete 16S rRNA gene sequence (1461 nucleotides) of strain GPA1^T^. Subsequently, the similarities between the 16S rRNA gene sequences of strain GPA1^T^ and its closely related type strains were calculated using EzBioCloud (http://www.ezbiocloud.net/identify) [15]. Alignment of the 16S rRNA gene sequences of strain GPA1^T^ and those of closely related type strains was conducted using the fast secondary-structure-aware infernal aligner (version 1.1.4) [16]. Phylogenetic trees based on the neighbour-joining (NJ), maximum-parsimony (MP), and maximum-likelihood (ML) algorithms, with bootstrap values (1000 replications), were reconstructed using mega11 software [17]. The Kimura two-parameter model, nearest-neighbour-interchange heuristic search method, and pairwise deletion options were applied to construct the NJ, MP, and ML trees, respectively.

**Fig. 1.**
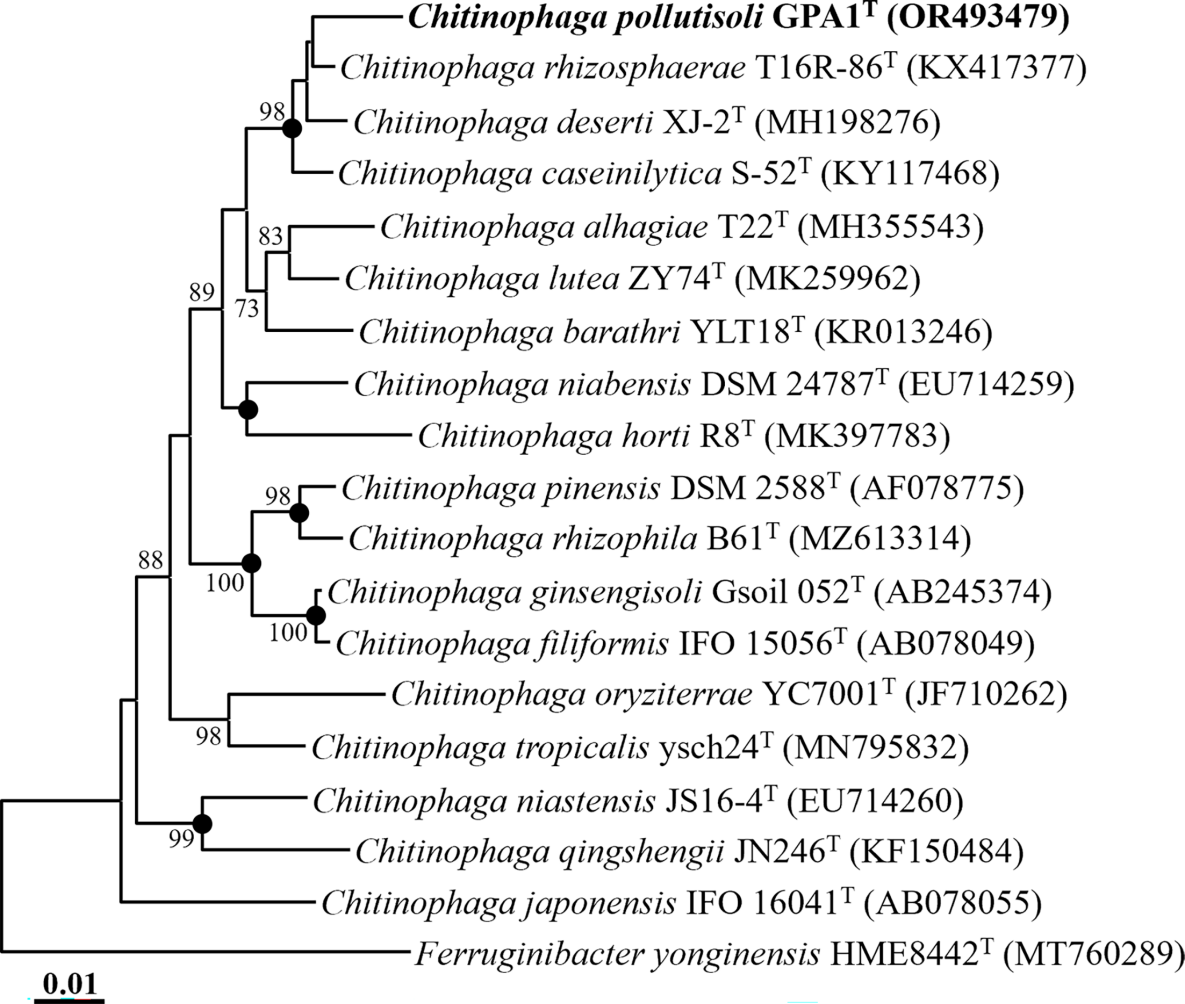
A neighbour-joining tree showing phylogenetic relationships between strain GPA1^T^ and their closely related taxa, based on 16S rRNA gene sequences. Only bootstrap values exceeding 70 % are indicated on the nodes as percentages, based on 1000 replicates. Filled circles (●) represent nodes that were also retrieved in the trees reconstructed using the maximum-likelihood and maximum-parsimony algorithms. *Ferruginibacter yonginensis* HME8442^T^ (MT760289) was employed as the outgroup. Bar, 0.01 substitutions per nucleotide.

Pairwise comparison of the 16S rRNA gene sequences revealed that strain GPA1^T^ shares the closest relationship with *C. rhizosphaerae* T16R-86^T^, *C. deserti* XJ-2^T^, and *C. caseinilytica* S-52^T^, exhibiting 98.6, 98.3, and 98.2 % 16S rRNA gene sequence similarities, respectively. Phylogenetic analysis, employing the NJ algorithm based on 16S rRNA gene sequences, demonstrated that strain GPA1^T^ forms a distinct phylogenetic lineage with *C. rhizosphaerae* T16R-86^T^, *C. deserti* XJ-2^T^, and *C. caseinilytica* S-52^T^ (Fig. 1). Furthermore, phylogenetic trees generated using the ML and MP algorithms further validated that strain GPA1^T^ clusters with *C. rhizosphaerae* T16R-86^T^, *C. deserti* XJ-2^T^, and *C. caseinilytica* S-52^T^ (Fig. S1, available in the online Supplementary Material). The collective results from comparative and phylogenetic analyses based on 16S rRNA gene sequences suggest that strain GPA1^T^ may represent a member of the genus *Chitinophaga*.

## Whole genome sequencing and genome-based phylogeny

For whole genome sequencing, genomic DNA from strain GPA1^T^ and *C. caseinilytica* KACC 19118^T^ was extracted from cells cultured in R2A broth using the Wizard Genomic DNA purification kit (Promega), following the manufacturer’s instructions. The extracted genomic DNA was sequenced in-house using the Oxford Nanopore MinION platform (ONT). Subsequently, sequencing reads obtained from Nanopore sequencing were *de novo*-assembled using Flye (version 2.9.1) [18]. The quality of the assembled genomes of strains GPA1^T^ and KACC 19118^T^ was assessed based on their completeness and contamination rates using CheckM2 software (version 1.0.2) [19].

A phylogenomic analysis of strains GPA1^T^ and KACC 19118^T^, along with closely related type strains, was conducted using the Genome Taxonomy Database Toolkit (GTDB-Tk), based on the concatenated protein sequences of 120 ubiquitous single-copy marker genes (bac120 marker set) [20]. An ML phylogenomic tree, supported by bootstrap values derived from 1000 replications, was reconstructed using mega 11 software. Average nucleotide identity (ANI) and digital DNA–DNA hybridization (dDDH) values among strains GPA1^T^ and KACC 19118^T^ and their closely related type strains were calculated using the Orthologous ANI Tool online (OAT, version 0.93.1; www.ezbiocloud.net/tools/orthoani) [21] and the Genome-to-Genome Distance Calculator (GGDC 3.0; https://ggdc.dsmz.de/ggdc.php) with formula 2 [22], respectively.

The *de novo* genome assembly of the MinION sequencing data for strains GPA1^T^ and KACC 19118^T^ resulted in complete genomes with sizes of 6078 and 6517 kb, respectively, consisting of one circular chromosome each. The 16S rRNA gene sequences identified within the genomes of strains GPA1^T^ and KACC 19118^T^ were found to be identical to those obtained by PCR-based sequencing. The completeness and contamination rates of the assembled genomes, as assessed by CheckM2, were 99.99 and 0.80 % for strain GPA1^T^, respectively, and 99.76 and 0.82 % for strain KACC 19118^T^, respectively. These values clearly met the criteria for generally high-quality genomes (completeness ≥90 % and contamination ≤10 %) [19].

**Fig. 2.**
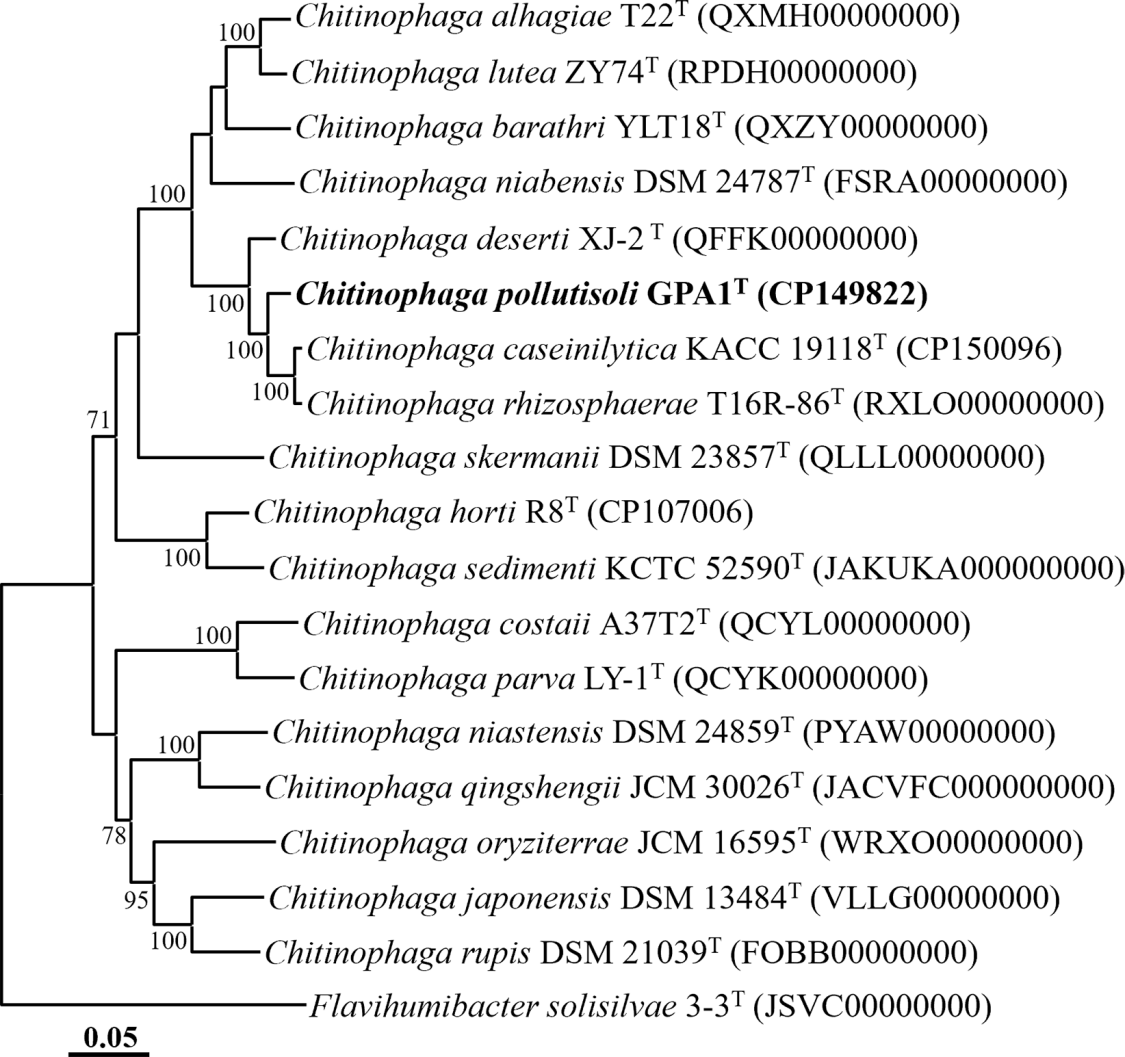
A phylogenomic tree showing the phylogenetic relationships between strain GPA1^T^ and closely related taxa, based on the concatenated amino acid sequences of 120 ubiquitous single-copy marker genes (bac120 marker set) of GTDB-Tk. Only bootstrap values exceeding 70 % are indicated on the nodes as percentages, based on 1000 replicates. *Flavihumibacter solisilvae* 3-3^T^ (JSVC00000000) was employed as the outgroup. Bar, 0.05 changes per amino acid position.

A phylogenomic analysis based on 120 single-copy marker protein sequences demonstrated that strain GPA1^T^ formed a robust phylogenetic lineage with *C. rhizosphaerae* T16R-86^T^ and *C. caseinilytica* KACC 19118^T^ within the genus *Chitinophaga*, supported by a 100 % bootstrap value (Fig. 2). The ANI and dDDH values between strain GPA1^T^ and closely related type strains, *C. rhizosphaerae* T16R-86^T^, *C. caseinilytica* KACC 19118^T^, and *C. deserti* XJ-2^T^, were determined to be 82.5, 82.3, and 79.5 %, and 25.9, 25.5, and 22.5 %, respectively (Table S1), which are significantly lower than the prokaryotic species delineation thresholds (ANI, 94–96 %; dDDH, 70 %) [23]. The results from the phylogenomic analysis and genome relatedness assessment strongly support the conclusion that strain GPA1^T^ represents a distinct novel species within the genus *Chitinophaga*.

## Genomic features

The whole genome sequences of strains GPA1^T^ and KACC 19118^T^ were submitted to GenBank and annotated using the NCBI Prokaryotic Genome Annotation Pipeline (www.ncbi.nlm.nih.gov/genome/annotation_prok/) [24]. Subsequently, carbohydrate-active enzymes (CAZys) were analysed using the dbCAN3 meta server (https://bcb.unl.edu/dbCAN2/) [25]. The genomes of strains GPA1^T^ and KACC 19118^T^ were predicted to contain 5197 and 5443 total genes, respectively, including 5080 and 5329 protein coding sequences, four rRNA operons (16, 23, and 5S rRNA genes), and 63 and 65 tRNA genes, respectively. The G+C contents calculated from the genomes of strains GPA1^T^ and KACC 19118^T^ were 53.8 and 55.0 mol%, respectively, aligning closely with the G+C contents observed in *Chitinophaga* species [3,10]. A summary and comparison of the genomic features of these strains with those of closely related type strains of the genus *Chitinophaga* are presented in Table 1, revealing generally similar genomic features among them.

**Table 1.** General genomic features of strains GPA1^T^ and closely related type strains of the genus *Chitinophaga* Strains: 1, GPA1^T^ (CP149822); 2, *C. rhizosphaerae* T16R-86^T^ (RXLO00000000); 3, *C. caseinilytica* KACC 19118^T^ (CP150096); 4, *C. deserti* XJ-2 ^T^ (QFFK00000000). The genomes of strain GPA1^T^ and *C. caseinilytica* KACC 19118^T^ were sequenced in this study.

Feature*	1	2	3	4
Genome status (no. of contigs)^†^	C (1)	D (28)	C (1)	D (26)
Total genome size (kb)	6078	6192	6517	6329
G+C content (mol%)	53.8	54.2	55.0	56.5
No. of total genes	5197	5119	5443	5118
No. of protein coding sequences	5080	5049	5329	5043
No. of total RNA genes	78	68	80	68
No. of tRNA genes	63	53	65	62
No. of rRNA genes	12	6	12	3
No. of total CAZy† genes	158	150	156	163
Glycoside hydrolases (GHs)	110	98	109	112
GH18	1	1	1	1
GH5	2	3	4	4
GH7, GH8, GH19, GH46, GH75, GH80	0	0	0	0
Glycosyl transferases	39	37	32	37
Polysaccharide lyases	5	5	6	6
Auxiliary activities	0	0	0	0
Carbohydrate esterases (CEs)	7	7	8	7
CE4	0	0	1	1
Carbohydrate-binding modules	2	4	3	2

†*The genomic features were analyzedanalysed using the NCBI prokaryotic genome annotation pipeline (www.ncbi.nlm.nih.gov/genome/annotation_prok/).

‡†C, complete; D, draft; CAZy, Ccarbohydrate-Aactive enZzyme.

Previous research studies have highlighted the chitin-degrading capabilities of members within the genus *Chitinophaga* [1,11, 26]. Thus, we conducted an extensive genome analysis to explore the distribution of CAZy-encoding genes, potentially involved in chitin degradation, in strain GPA1^T^ and closely related *Chitinophaga* strains. Various CAZys have been implicated in chitin degradation, including chitinases (GH18 or GH19) and chitin deacetylases (CE4, GH5, GH7, or GH8), which break down chitin into *N*-acetyl-glucosamine and chitosan, respectively. Additionally, chitosanases (GH46, GH75, or GH80) can further degrade chitin into *N*-glucosamine [27]. The CAZy gene analysis unveiled that strain GPA1^T^ and the closely related reference strains possessed a diverse array of CAZy genes spanning the major six categories—glycoside hydrolases (GHs), glycosyltransferases, polysaccharide lyases, carbohydrate esterases (CEs), auxiliary activities, and carbohydrate-binding modules (Table 1). In strain GPA1^T^, a diverse set of 158 putative CAZy coding genes, including chitin degradation-related genes such as GH5 and GH18, were identified. These CAZy coding genes were similarly found in reference strains of the genus *Chitinophaga* (Table 1), indicating their potential for decomposing various carbohydrates. However, despite the presence of these genes, our chitin hydrolysis test did not reveal any chitin hydrolysing activity in strain GPA1^T^ or the reference type strains. These results suggest that the chitin-degrading genes identified in strain GPA1^T^ and three reference strains may not be sufficient for chitin degradation. Specifically, the absence of GH7, GH8, GH19, GH46, GH75, GH80, and CE4 genes in strain GPA1^T^ appears to be a significant reason for the lack of chitin hydrolysis activity. Additionally, although strain GPA1^T^ was isolated from an enriched culture for the isolation of PA-degrading bacteria, it exhibited no ability to degrade PA, and PA degradation-related genes were also not identified in its genome. These results suggest that strain GPA1^T^ may rely on other PA-degrading microbes or utilize their PA degradation by-products for its survival in the enrichment culture using PA.

## Morphology and physiology

Growth of strain GPA1^T^ was assessed on various bacteriological agar media, including R2A agar, tryptic soy agar (MBcell), nutrient agar (MBcell), marine agar (MBcell), and Luria-Bertani (LB) agar (MBcell), at 30 °C for 2 days to determine the optimal agar medium. Subsequently, the growth of strain GPA1^T^ and reference strains was examined at different temperatures (5–45 °C at 5 °C intervals) and pH values (pH 4.0–11.0 at 1.0 pH unit intervals at 30 °C) on R2A agar and R2A broth for 2 days, respectively. R2A broth media with desired pH levels were prepared using sodium citrate (pH 4.0–5.0), sodium phosphate (pH 6.0–8.0), and sodium carbonate–bicarbonate (pH 9.0–11.0) buffers, followed by pH readjustment post-sterilization if necessary. Salt tolerance of strain GPA1^T^ and reference strains was assessed in R2A broth with varying NaCl concentrations (0–5.0 % at 0.5 % intervals, w/v) at 30 °C for 2 days. Anaerobic growth of strain GPA1^T^ was evaluated by streaking on R2A agar and incubating for 21 days at 30 °C under anaerobic conditions created using the GasPak Plus system (BBL).

The following physiological and biochemical tests were conducted using cells grown on R2A agar at 30 °C for 2 days. Cellular morphology and flagella motility were examined using a phase-contrast microscope (Zeiss Axio Scope. A1, Carl Zeiss). Furthermore, for a detailed examination of cell morphology, size, and flagella, cells were affixed to formvar-coated copper grids, negatively stained with 2 % (w/v) uranyl acetate (Sigma-Aldrich) for 15 s, and subsequently examined under a transmission electron microscope (JEM-1010, jeol). Gliding motility was estimated on R2A agar containing 0.3 % agar. Gram staining was performed using a Gram stain kit from bioMérieux, following the manufacturer’s instructions. Catalase and oxidase activities were assessed by observing oxygen bubble production in a 3 % (v/v) aqueous hydrogen peroxide solution (Junsei) and the oxidation of 1 % (w/v) tetramethyl-*p*-phenylenediamine (Merck), respectively. Hydrolysis of casein (1.0 % skimmed milk, w/v), starch (1.0 %), tyrosine (0.5 %), Tween 20 (1.0 %), Tween 80 (1.0 %), aesculin (0.1 %, w/v), and chitin (0.1 %) was assessed on R2A agar, following previously established protocols [28]. Methyl red and Voges–Proskauer tests were conducted in R2A broth supplemented with 0.5 % (w/v) glucose, following the protocol described by Smibert and Krieg [28]. Hydrogen sulphide production was assessed using sulphide indole motility medium, as described previously [29]. Minimum inhibitory concentration (MIC) of clindamycin, ampicillin, streptomycin, gentamicin, kanamycin, tetracycline, chloramphenicol, vancomycin, and erythromycin for strain GPA1^T^ was measured on R2A agar at 30 °C using Liofilchem test strips. Additionally, the MIC of rifampin for strain GPA1^T^ was determined on R2A agar with varying concentrations of rifampin at 30 °C. Additional biochemical features and enzymatic activities were evaluated using the API 20NE kit from bioMérieux, following the manufacturer’s provided instructions.

Strain GPA1^T^ exhibited optimal growth on R2A agar, with favourable growth also observed on tryptic soy agar, but failed to grow on nutrient agar, marine agar, and LB agar. Cells of strain GPA1^T^ were Gram-stain-negative, non-motile short rods, 0.5–0.6 µm wide, and 0.8–1.0 µm long (Fig. S2). Under anaerobic conditions, strain GPA1^T^ exhibited growth after 21 days, indicating its facultative aerobic nature. Strain GPA1^T^ exhibited MIC values for the following antibiotics: clindamycin (32 µg ml^−1^), ampicillin (>256 µg ml^−1^), streptomycin (128 µg ml^−1^), gentamicin (48 µg ml^−1^), kanamycin (> 256 µg ml^−1^), tetracycline (24 µg ml^−1^), chloramphenicol (>256 µg ml^−1^), vancomycin (16 µg ml^−1^), erythromycin (>256 µg ml^−1^), and rifampin (1 µg ml^−1^). The strain exhibited a high MIC for ampicillin, but a low MIC for rifampin although genomic analysis revealed that strain GPA1^T^ harbours resistance genes for *β*-lactams (*bla*) and rifampin (*rphB*) antibiotics. In addition, despite not identifying resistance genes in its genome, strain GPA1^T^ showed high MICs for clindamycin, streptomycin, gentamicin, kanamycin, tetracycline, chloramphenicol, vancomycin, and erythromycin. Many phenotypic, physiological, and biochemical properties of strain GPA1^T^, including flagellum motility, the activity of catalase and *β*-galactosidase, and assimilation of d-glucose, d-mannose, *N*-acetylglucosamine, and maltose, as well as aesculin hydrolysis, were similar to those of three reference strains (Table 2). However, some properties such as oxidase activity, assimilation of potassium gluconate, adipic acid, malic acid, and citric acid, and hydrolysis of casein and tyrosine, allowed for its differentiation from closely related *Chitinophaga* species.

**Table 2.** Comparison of phenotypic characteristics of strain GPA1^T^ and closely related type strains of the genus *Chitinophaga* Strains: 1, GPA1^T^ (this study); 2, *C. rhizosphaerae* KACC 18790^T^ [34]; 3, *C. caseinilytica* KACC 19118^T^ [33]; 4, *C. deserti* KCTC 62443^T^ [35]. All strains are positive for the following characteristics: activity* of catalase and *β*-galactosidase, hydrolysis* of aesculin, and assimilation* of d-mannose, *N*-acetylglucosamine, and maltose. All strains are negative for the following characteristics: Gram-staining, flagellum motility, indole production, nitrate reduction, glucose fermentation, activity* of arginine dihydrolase and urease, Voges−Proskauer test, H_2_S production, hydrolysis of Tween 20, starch, and chitin, and assimilation* of l-arabinose, d-mannitol, capric acid, and phenylacetic acid. +, Positive; –, negative; w, weakly positive.

Characteristic	1	2	3	4
Isolation source	Plastic waste landfill soil	Rhizosphere soil	Forest soil	Desert soil
Colony colour	Yellow	Yellow	Golden-yellow	Light yellow
Cell shape	Rod	Rod or filamentous	Rod	Rod
Growth range of:*				
Temperature (optimum; °C)	15–40 (30)	15–40 (35); 10–37 (28)†	15–40 (30–35); 20–37 (28–32)†	15–40 (30); 10–37 (28–32)†
pH (optimum)	6.0–9.0 (7.0–8.0)	6.0–9.0 (7.0–8.0); 6.0–9.0 (7.0)†	6.0–9.0 (8.0); 6.5–11.0 (7.0–9.5)†	6.0–9.0 (8.0); 6.0–11.0 (7.0)†
NaCl (%, w/v)	0–2.5	0–2.5; 0–2.0†	0–2.5; 0–3.0†	0–2.5; 0–1.0†
Oxidase activity*	–	w	w	–
Methyl red test	–	–	–	–; +†
Hydrolysis of:*				
Casein	–	w	+	+; –†
Tyrosine	–	+; –†	w; –†	–
Gelatin	–	–; +†	–	–
Tween 80	–	–	–; +†	–; +†
Assimilation of:*				
d-Glucose	+	+	+	+; –†
Potassium gluconate	–	w; –†	w; –†	w; –†
Adipic acid	–	w; –†	–	w; –†
Malic acid	w	w; –†	–	w; –†
Citric acid	w	w; –†	–	–
Major polar lipids‡	PE, AL, 2GLs, 4Ls	PE, APL, PL, 5ALs, 2Ls	PE, DPG, 2APLs, AL, 4Ls	PE, 3ALs, GL, 5Ls

*These data were obtained from this study under the same conditions.

‡†The data presented here are inconsistent with the results reported in the reference studies, and the second data represent the results from previous studies.

†‡PE, phosphatidylethanolamine; DPG, diphosphatidylglycerol; APL, unidentified aminophospholipid; PL, unidentified phospholipid; AL, unidentified aminolipid; GL, unidentified glycolipid; L, unidentified lipid.

## Chemotaxonomic characteristics

The respiratory isoprenoid quinones of strain GPA1^T^ were extracted from cells cultured in R2A broth for 2 days at 30 °C, following the protocol outlined by Minnikin *et al*. [30]. The extracted quinones were analysed using an HPLC system (model LC-20A, Shimadzu) equipped with a reversed-phase column (250×4.6 mm, Kromasil, Akzo Nobel) and a diode array detector (SPD-M20A, Shimadzu). Methanol–isopropanol (2 : 1, v/v) was used as the eluent at a flow rate of 1 ml min^−1^. For the analysis of cellular fatty acids, strain GPA1^T^ and three reference strains were aerobically cultivated in R2A broth at 30 °C until reaching the exponential growth phase (optical density, OD600=0.7–0.8). Cellular fatty acids were saponified, methylated, and extracted following the standard procedure outlined by midi (Sherlock Microbial Identification System, version 6.2B). Fatty acid methyl esters were analysed using a gas chromatograph (Hewlett Packard 6890) and identified with the RTSBA6 database of the Microbial Identification System (Sherlock version 6.0B) [31]. The polar lipids of strain GPA1^T^ were extracted from cells harvested during the exponential growth phase and analysed by two-dimensional TLC, following the procedure described by Minnikin *et al*. [32]. Various reagents were employed for the identification of different polar lipids: 10 % ethanolic molybdophosphoric acid (for total polar lipids), ninhydrin (for aminolipids), Dittmer–Lester (for phospholipids), and *α*-naphthol/sulphuric acid (for glycolipids). The presence of PE in strain GPA1^T^ was confirmed using a standard PE compound purchased from Sigma-Aldrich.

MK-7 was identified as the sole respiratory quinone in strain GPA1^T^, aligning well with findings in other *Chitinophaga* members [3,10,32]. Analysis of cellular fatty acids revealed iso-C_15 : 0_, C_16 : 1_* ω*5*c*, and iso-C_17 : 0_ 3-OH as the major fatty acids (>10.0 % of total fatty acids) in strain GPA1^T^, consistent with three reference strains (Table S2). While the overall fatty acid profiles of strain GPA1^T^ and reference *Chitinophaga* members were generally similar, differences in the proportions of fatty acids such as C_12 : 0_, iso-C_10 : 0_, iso-C_15 : 0_ 2-OH, and C_16 : 1_* ω*11*c* distinguished strain GPA1^T^ from closely related *Chitinophaga* species. Strain GPA1^T^ contained PE, an unidentified aminolipid, two unidentified glycolipids, and four unidentified lipids as major polar lipids (Fig. S3). The presence of PE as a major polar lipid in strain GPA1^T^ was consistent with observations in closely related *Chitinophaga* species [33,34].

## Taxonomic conclusion

The phylogenetic inference and genomic relatedness, as well as the phenotypic, biochemical, and chemotaxonomic characteristics, support the notion that strain GPA1^T^ represents a novel species of the genus *Chitinophaga*, for which the name *Chitinophaga pollutisoli* sp. nov. is proposed.

## Description of *Chitinophaga pollutisoli* sp. nov.

*Chitinophaga pollutisoli* (pol.lu.ti.so′li. L. perf. part. *pollutus*, polluted; L. neut. n. *solum*, soil; N.L. gen. n. *pollutisoli*, of polluted soil).

Colonies on R2A agar are yellow, circular, and convex with entire margin. Cells are Gram-stain-negative, facultative aerobic, and non-motile short rods. Gliding motility is negative. Growth occurs at 15–40 °C (optimum, 30 °C) and pH 6.0–9.0 (optimum, pH 7.0–8.0) and in the presence of 0–2.5 % (w/v) NaCl (optimum, 0 %). Catalase-positive and oxidase-negative. Nitrate is not reduced to nitrite. Methyl red test, Voges–Proskauer test, and H_2_S production are negative. Aesculin is hydrolysed, but casein, l-tyrosine, Tween 20, Tween 80, starch, gelatin, and chitin are not. Glucose fermentation and indole production are negative. *β*-Galactosidase activity is positive, but arginine dihydrolase and urease activities are negative. Assimilation of d-glucose, d-mannose, *N*-acetylglucosamine, maltose, malic acid (weakly), and citric acid (weakly) is positive, but that of l-arabinose, d-mannitol, capric acid, phenylacetic acid, potassium gluconate, and adipic acid is negative. MK-7 is the sole respiratory quinone, and PE is identified as a major polar lipid. The major fatty acids (>10 % of the total fatty acids) are iso-C_15 : 0_, C_16 : 1_* ω*5*c*, and iso-C_17 : 0_ 3-OH.

The type strain is GPA1^T^ (=KACC 23415^T^=JCM 36644^T^), isolated from contaminated sediment collected from a stream flowing through an industrial complex in Incheon, Republic of Korea. The genome size and DNA G+C content of the type strain are 6078 kb and 53.8 mol% (calculated from the whole genome sequence), respectively. The GenBank accession numbers for the 16S rRNA gene and genome sequences of strain GPA1^T^ are OR493479 and CP149822, respectively.

## supplementary material

10.1099/ijsem.0.006447 Uncited Supplementary Material 1.

## Abbreviations

ANI: average nucleotide identity
CAZy: carbohydrate-active enzyme
dDDH: digital DNA–DNA hybridization
MIC: minimum inhibitory concentration
MK-7: menaquinone-7
ML: maximum-likelihood
MP: maximum-parsimony
MSB: minimal salts basal
NJ: neighbour-joining
PA: phthalic acid
PE: phosphatidylethanolamine
R2A: Reasoner's 2A

## References

[1] Sangkhobol V, Skerman VBD (1981). *Chitinophaga*, a new genus of chitinolytic myxobacteria. Int J Syst Bacteriol.

[2] Kämpfer P, Lodders N, Falsen E (2011). *Hydrotalea flava* gen. nov., sp. nov., a new member of the phylum *Bacteroidetes* and allocation of the genera *Chitinophaga*, *Sediminibacterium*, *Lacibacter*, *Flavihumibacter*, *Flavisolibacter*, *Niabella*, *Niastella*, *Segetibacter*, *Parasegetibacter*, *Terrimonas*, *Ferruginibacter*, *Filimonas* and *Hydrotalea* to the family *Chitinophagaceae* fam. nov. Int J Syst Evol Microbiol.

[3] Trinh NH, Kim J (2023). *Chitinophaga nivalis* sp. nov., isolated from forest soil in Pyeongchang, Republic of Korea. Int J Syst Evol Microbiol.

[4] Han DM, Baek JH, Choi DG, Jin MS, Jeon CO (2023). *Chitinophaga horti* sp. nov., isolated from garden soil. Curr Microbiol.

[5] He SW, Ma R, Zhao YY, An L, Huang JH (2022). *Chitinophaga hostae* sp. nov., isolated from the rhizosphere soil of Hosta plantaginea. Int J Syst Evol Microbiol.

[6] Onouye TC, Busse HJ, Prescott RD, Darris MK, Donachie SP (2023). *Chitinophaga pendula*, sp. nov., from an air conditioner condensate drain line. Int J Syst Evol Microbiol.

[7] Jin D, Kong X, Wang J, Sun J, Yu X (2018). *Chitinophaga caeni* sp. nov., isolated from activated sludge. Int J Syst Evol Microbiol.

[8] Li L, Sun L, Shi N, Liu L, Guo H (2013). *Chitinophaga cymbidii* sp. nov., isolated from *Cymbidium goeringii* roots. Int J Syst Evol Microbiol.

[9] Zong Y, Wu M, Liu X, Jin Y, Wang G (2019). *Chitinophaga lutea* sp. nov., isolated from arsenic-contaminated soil. Int J Syst Evol Microbiol.

[10] Yasir M, Chung EJ, Song GC, Bibi F, Jeon CO (2011). *Chitinophaga eiseniae* sp. nov., isolated from vermicompost. Int J Syst Evol Microbiol.

[11] Kämpfer P, Young C-C, Sridhar KR, Arun AB, Lai WA (2006). Transfer of [*Flexibacter*] *sancti*, [*Flexibacter*] *filiformis*, [*Flexibacter*] *japonensis* and [*Cytophaga*] *arvensicola* to the genus *Chitinophaga* and description of *Chitinophaga skermanii* sp. nov. Int J Syst Evol Microbiol.

[12] Stanier RY, Palleroni NJ, Doudoroff M (1966). The aerobic pseudomonads: a taxonomic study. Microbiology.

[13] Lee Y, Jeon CO (2018). *Paraburkholderia aromaticivorans* sp. nov., an aromatic hydrocarbon-degrading bacterium, isolated from gasoline-contaminated soil. Int J Syst Evol Microbiol.

[14] Kim KR, Kim JM, Lee JK, Han DM, Hao L (2023). *Dyadobacter pollutisoli* sp. nov., isolated from plastic waste landfill soil. Int J Syst Evol Microbiol.

[15] Yoon S-H, Ha S-M, Kwon S, Lim J, Kim Y (2017). Introducing EzBioCloud: a taxonomically united database of 16S rRNA gene sequences and whole-genome assemblies. Int J Syst Evol Microbiol.

[16] Nawrocki EP, Eddy SR (2013). Infernal 1.1: 100-fold faster RNA homology searches. Bioinformatics.

[17] Tamura K, Stecher G, Kumar S (2021). MEGA11: Molecular Evolutionary Genetics Analysis version 11. Mol Biol Evol.

[18] Kolmogorov M, Yuan J, Lin Y, Pevzner PA (2019). Assembly of long, error-prone reads using repeat graphs. Nat Biotechnol.

[19] Chklovski A, Parks DH, Woodcroft BJ, Tyson GW (2023). CheckM2: a rapid, scalable and accurate tool for assessing microbial genome quality using machine learning. Nat Methods.

[20] Chaumeil PA, Mussig AJ, Hugenholtz P, Parks DH (2019). GTDB-Tk: a toolkit to classify genomes with the genome taxonomy database. Bioinformatics.

[21] Lee I, Ouk Kim Y, Park S-C, Chun J (2016). OrthoANI: an improved algorithm and software for calculating average nucleotide identity. Int J Syst Evol Microbiol.

[22] Meier-Kolthoff JP, Auch AF, Klenk HP, Göker M (2013). Genome sequence-based species delimitation with confidence intervals and improved distance functions. BMC Bioinform.

[23] Chun J, Oren A, Ventosa A, Christensen H, Arahal DR (2018). Proposed minimal standards for the use of genome data for the taxonomy of prokaryotes. Int J Syst Evol Microbiol.

[24] Tatusova T, DiCuccio M, Badretdin A, Chetvernin V, Nawrocki EP (2016). NCBI prokaryotic genome annotation pipeline. Nucleic Acids Res.

[25] Zhang H, Yohe T, Huang L, Entwistle S, Wu P (2018). dbCAN2: a meta server for automated carbohydrate-active enzyme annotation. Nucleic Acids Res.

[26] Lu Z, Kvammen A, Li H, Hao M, Inman AR (2023). A polysaccharide utilization locus from *Chitinophaga pinensis* simultaneously targets chitin and β-glucans found in fungal cell walls. mSphere.

[27] De Tender C, Mesuere B, Van der Jeugt F, Haegeman A, Ruttink T (2019). Peat substrate amended with chitin modulates the N-cycle, siderophore and chitinase responses in the lettuce rhizobiome. Sci Rep.

[28] Smibert RM, Krieg NR, Gerhardt P, Murray RGE, Wood WA, Krieg NR (1994). Methods for General and Molecular Bacteriology.

[29] Vashist H, Sharma D, Gupta A (2013). A review on commonly used biochemical test for bacteria. Innovare J Life Sci.

[30] Minnikin DE, O’Donnell AG, Goodfellow M, Alderson G, Athalye M (1984). An integrated procedure for the extraction of bacterial isoprenoid quinones and polar lipids. J Microbiol Methods.

[31] Sasser M (1990). MIDI Technical Note.

[32] Minnikin DE, Patel PV, Alshamaony L, Goodfellow M (1977). Polar lipid composition in the classification of nocardia and related bacteria. Int J Syst Bacteriol.

[33] Dahal RH, Kim J (2018). *Chitinophaga caseinilytica* sp. nov., a casein hydrolysing bacterium isolated from forest soil. Arch Microbiol.

[34] Kim S-J, Cho H, Ahn J-H, Weon H-Y, Joa J-H (2017). *Chitinophaga rhizosphaerae* sp. nov., isolated from rhizosphere soil of a tomato plant. Int J Syst Evol Microbiol.

[35] Kong XK, Chen D, Huang JW, Cheng XK, Jiang JD (2019). *Chitinophaga deserti* sp. nov., isolated from desert soil. Int J Syst Evol Microbiol.

