# Supplementary material for: Chitinophaga pollutisoli sp. nov., isolated from contaminated sediment
Source: Int J Syst Evol Microbiol. 2024 Jul 4;74(7):006447. doi: 10.1099/ijsem.0.006447 (PMC11316580; doi:10.1099/ijsem.0.006447)
Supplement: Uncited Supplementary Material 1. [file ijsem-74-06447-s001.pdf]

Supplementary Information

Fig. S1. Maximum-likelihood (A) and maximum-parsimony (B) trees showing the phylogenetic relationships between strain GPA1^T and closely related taxa, based on 16S rRNA gene sequences. Only bootstrap values exceeding 70% are indicated on the nodes as percentages from 1000 replicates. *Ferruginibacter yonginensis* HME8442^T (MT760289) was used as the outgroup. Scale bars in panels A and B denote substitutions per nucleotide and nucleotide substitutions over the whole sequences, respectively.

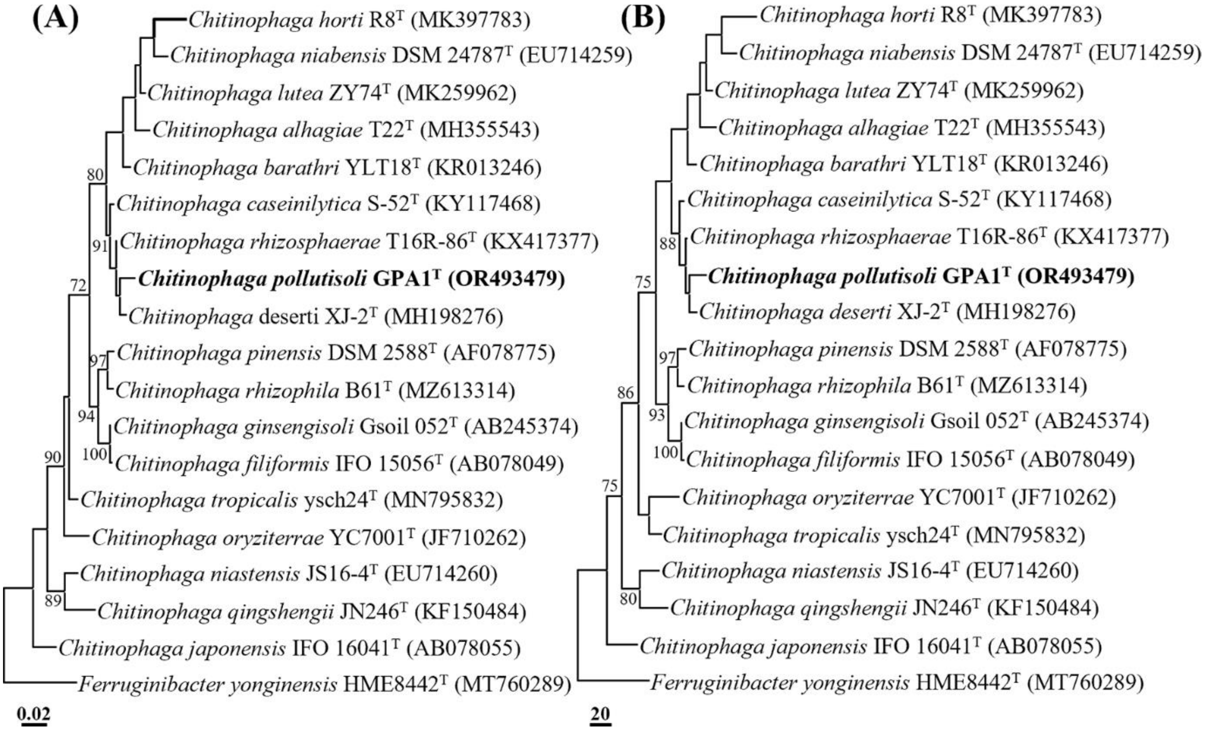

Fig. S2. A transmission electron micrograph showing the general cellular morphology of strain GPA1^T after 2 days of cultivation on R2A agar at 30°C. Cells were negatively stained with 2% uranyl acetate. Scale bar, 500 nm.

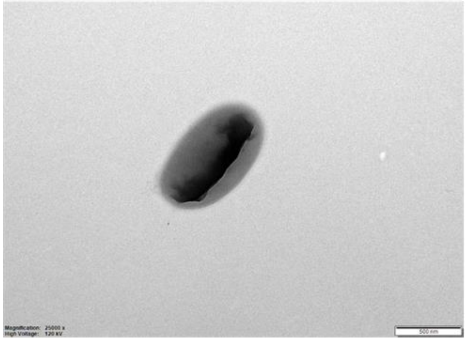

Fig. S3. Two-dimensional thin-layer chromatograms (TLC) showing the polar lipids of strain GPA1^T. Solvent systems: (I) chloroform-methanol-water (65:25:4, v/v/v) and (II) chloroform-acetic acid-methanol-water (80:15:12:4, v/v/v/v). The TLC plates were sprayed with 10% ethanolic molybdophosphoric acid (A), ninhydrin (B), Dittmer-Lester (C), and *α*-naphthol/sulfuric acid (D) reagents for the detection of total polar lipids, aminolipids, phospholipids, and glycolipids, respectively. PE, phosphatidylethanolamine; AL, unidentified aminolipid; GL1–2, unidentified glycolipids; L1–4, unidentified lipids.

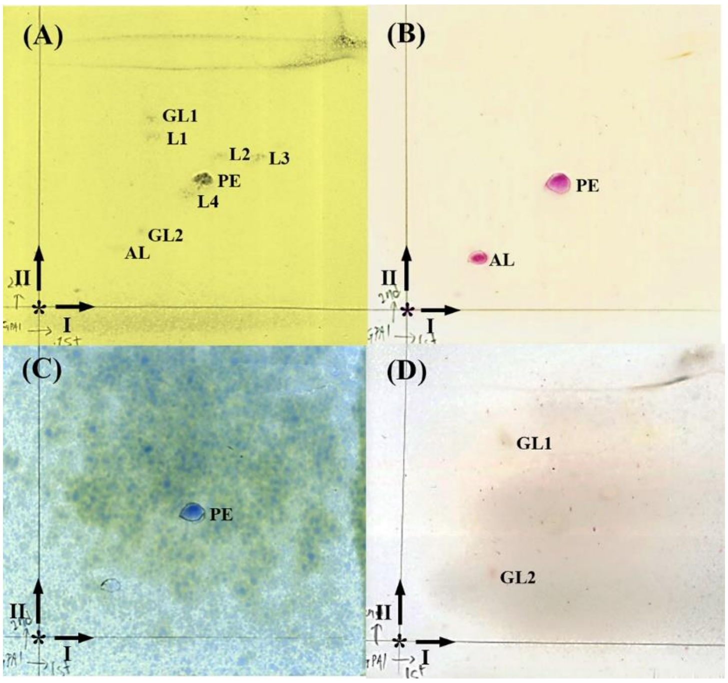

Table S1. Genome relatedness among strain GPA1^T and closely related type strains of the genus *Chitinophaga*

Taxa: 1, Strain GPA1^T (CP149822); 2, *C. rhizosphaerae* T16R-86^T (RXLO00000000); 3. *C. caseinilytica* KACC 19118^T (CP150096); 4. *C. deserti* XJ-2^T (QFFK00000000).

		dDDH† value (%)			
		**1**	**2**	**3**	**4**
ANI† value (%)	**1**	–	25.9	25.5	22.5
	**2**	82.5	–	50.4	22.3
	**3**	82.3	93.0	–	22.1
	**4**	79.5	79.3	79.2	–

†ANI, average nucleotide identity; dDDH, digital DNA-DNA hybridization.

Table S2. Comparison of cellular fatty acid compositions (%) of strain GPA1^T and closely related type strains of the genus *Chitinophaga*

Taxa: 1, strain GPA1^T; 2, *C. rhizosphaerae* KACC 18790^T; 3. *C. caseinilytica* KACC 19118^T; 4. *C. deserti* KCTC 62443^T. All data were obtained from this study. Data are expressed as percentages of the total fatty acids, and fatty acids constituting less than 1.0% in all strains are not shown. Major components (> 10.0%) are highlighted in bold. tr, trace amount (< 1.0%); –, not detected.

**Fatty acid**	**1**	**2**	**3**	**4**
Saturated:				
$C_{12:0}$	–	1.5	tr	tr
$C_{14:0}$	2.0	1.3	1.4	1.4
$C_{16:0}$	tr	1.1	tr	tr
Unsaturated:				
$C_{15:1}$ $\omega 5c$	2.0	1.1	2.5	2.5
$C_{16:1}$ $\omega 11c$	–	1.1	tr	tr
$C_{16:1}$ $\omega 5c$	**23.1**	**17.8**	**19.1**	**23.0**
Branched:				
iso-$C_{10:0}$	tr	1.8	tr	–
iso-$C_{15:0}$	**44.4**	**36.0**	**40.2**	**40.5**
iso-$C_{16:0}$	tr	1.6	1.9	1.0
Hydroxy:				
iso-$C_{15:0}$ 3-OH	5.7	6.9	6.3	5.3
iso-$C_{15:0}$ 2-OH	1.8	1.8	–	–
iso-$C_{16:0}$ 3-OH	–	–	–	1.1
iso-$C_{17:0}$ 3-OH	**10.8**	**15.9**	**13.4**	**12.6**
Summed feature*:				
3 ($C_{16:1}$ $\omega 7c$ and/or $C_{16:1}$ $\omega 6c$)	2.8	7.4	5.4	4.7

*Summed features are fatty acids that cannot be resolved reliably from another fatty acid using the chromatographic conditions chosen. The MIDI system groups these fatty acids together as one feature with a single percentage of the total.
